# Expression and functional analysis of *TaASY1 *during meiosis of bread wheat (*Triticum aestivum*)

**DOI:** 10.1186/1471-2199-8-65

**Published:** 2007-08-04

**Authors:** Scott A Boden, Nadim Shadiac, Elise J Tucker, Peter Langridge, Jason A Able

**Affiliations:** 1 Molecular Plant Breeding Cooperative Research Centre, School of Agriculture, Food & Wine, The University of Adelaide, Waite Campus, PMB1, Glen Osmond, South Australia, 5064, Australia; 2 Australian Centre for Plant Functional Genomics, School of Agriculture, Food & Wine, The University of Adelaide, Waite Campus, PMB1, Glen Osmond, South Australia, 5064, Australia; 3 Institute of Biology III, University of Freiburg, Schänzlestraße 1, 79104 Freiburg, Germany

## Abstract

**Background:**

Pairing and synapsis of homologous chromosomes is required for normal chromosome segregation and the exchange of genetic material via recombination during meiosis. Synapsis is complete at pachytene following the formation of a tri-partite proteinaceous structure known as the synaptonemal complex (SC). In yeast, HOP1 is essential for formation of the SC, and localises along chromosome axes during prophase I. Homologues in *Arabidopsis *(*AtASY1*), *Brassica *(*BoASY1*) and rice (*OsPAIR2*) have been isolated through analysis of mutants that display decreased fertility due to severely reduced synapsis of homologous chromosomes. Analysis of these genes has indicated that they play a similar role to HOP1 in pairing and formation of the SC through localisation to axial/lateral elements of the SC.

**Results:**

The full length wheat cDNA and genomic clone, *TaASY1*, has been isolated, sequenced and characterised. *TaASY1 *is located on chromosome Group 5 and the open reading frame displays significant nucleotide sequence identity to *OsPAIR2 *(84%) and *AtASY1 *(63%). Transcript and protein analysis showed that expression is largely restricted to meiotic tissue, with elevated levels during the stages of prophase I when pairing and synapsis of homologous chromosomes occur. Immunolocalisation using transmission electron microscopy showed *Ta*ASY1 interacts with chromatin that is associated with both axial elements before SC formation as well as lateral elements of formed SCs.

**Conclusion:**

*TaASY1 *is a homologue of *ScHOP1*, *AtASY1 *and *OsPAIR2 *and is the first gene to be isolated from bread wheat that is involved in pairing and synapsis of homologous chromosomes.

## Background

Meiosis is obligatory for sexual reproduction and is comprised of one round of DNA replication followed by two rounds of cell division. There are three key processes that occur during early meiosis which are responsible for the juxtaposition of homologous chromosomes required for successful production of haploid gametes, namely chromosome pairing, recombination and chromosome synapsis. Studies investigating the molecular nature of homologous chromosome pairing have revealed a complex relationship between these three processes.

Complexity is particularly pronounced in polyploid organisms such as the allohexaploid bread wheat (*Triticum aestivum*). Bread wheat contains seven groups of chromosomes which are derived from three diploid progenitor species (AABBDD; 2n = 6× = 42). Despite the genome complexity of this important crop, each chromosome will pair only with its homologue, despite the potential for pairing with an equivalent chromosome from one of the other two related or homoeologous genomes. Extensive cytological analysis of chromosome dynamics during early meiosis in normal bread wheat and mutants such as *Ph1 *and *Ph2 *(pairing homoeologous), which display reduced specificity of chromosome pairing to homologues, have provided valuable information on the control of chromosome pairing in this organism [1-7]. However, to date there are no individual proteins that have been identified and characterised from bread wheat that have been shown to have a role in homologous chromosome pairing and synapsis.

Pairing of homologous chromosomes is closely followed by synapsis through the formation of a proteinaceous structure referred to as the synaptonemal complex (SC) [8-10]. The evolutionary conservation of the SC within sexually reproducing organisms and its demonstrated association with recombination indicates a fundamental and critical role during meiosis I (for comprehensive reviews on the biology of the SC refer to [11-13]). The SC is composed of three components: the axial/lateral elements, transverse filaments and a dense central element. While several genes encoding SC and SC-associated proteins including ZIP1 [14] and SCP1 [15] have been isolated and characterised in both yeast and mammals since the discovery of this structure 50 years ago, it has only been recently that the first SC plant specific protein was reported [16], even though this structure has been comprehensively dissected cytologically. The slow progress in plants is mainly due to limited sequence conservation of SC proteins from various eukaryotic species, as reflected in a study of ZYP1 from *Arabidopsis thaliana*, which shares only 18 and 20% sequence identity to ZIP1 and SCP1 from yeast and mouse respectively [16]. This problem is being overcome through two approaches. Reverse genetics is being used in Arabidopsis and rice to identify genes involved in chromosome synapsis by analysing mutants that display an abnormal synaptic phenotype during early meiosis, and additionally, an *in silico *screening of databases is used to identify proteins that contain secondary structures that are conserved amongst known SC proteins [16,17].

One class of meiotic mutants used to identify genes that code for components of the SC in plants are termed asynaptic. These mutants are typically characterised as being defective in homologous chromosome synapsis, from which other defects follow, including dramatically increased frequency of univalents at pachytene and reduced fertility [18-21]. Several such genes have been reported in the literature including *HOP1 *from yeast (*Saccharomyces cerevisiae*) [22,23], *ASY1 *from Arabidopsis and *Brassica oleracea *[19,24] and *PAIR2 *from rice (*Oryza sativa*) [20,25]. In addition, the location and activity of ASY1 orthologues during meiosis in maize and rye has been investigated using the *At*ASY1 antibody in studies of mutants displaying abnormal chromosomal morphology during prophase I, which also supports results obtained from Arabidopsis and rice [26,27]. A common feature of the three characterised asynaptic genes is the presence of a HORMA domain (Hop1, Rev7, MAD2) [28] which appears to facilitate direct interaction of proteins containing this domain with chromatin during mitosis and meiosis, including chromatin associated with DNA adducts and DNA double stranded breaks [23,24,28,29].

In an attempt to further understand the mechanism of pairing/synapsis of homologous chromosomes during meiosis in bread wheat, we have characterised the expression and protein localisation of the wheat orthologue of *ASY1 *of Arabidopsis, *TaASY1*. This gene shares significant sequence identity and similar features (including the presence of a HORMA domain) to previously characterised asynaptic genes including the Arabidopsis, *Brassica *and rice orthologues and is likely to play a pivotal role during meiosis in bread wheat.

## Results

### *TaASY1 *is a wheat orthologue of *AtASY1*, *BoASY1 *and rice *PAIR2 *and is located on wheat chromosome group 5

**Figure 1:**
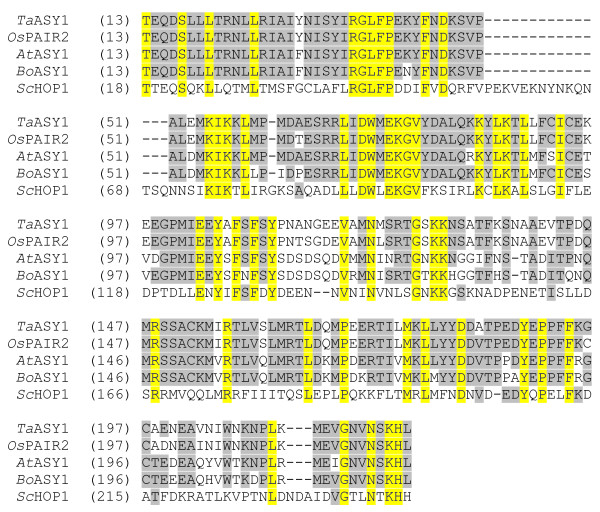
**Alignment and comparison of the deduced amino acid sequences within the HORMA domains of *Ta*ASY1, *Os*PAIR2 (*O. sativa*), *At*ASY1 (*A. thaliana*), *Bo*ASY1 (*B. oleracea*) and *Sc*HOP1 (*S.cerevisiae*)**. Conserved amino acid residues are highlighted in blue with a yellow background, while conserved plant sequence residues are highlighted in black with a grey background.

Analysis of the complete open reading frame (ORF) of *TaASY1 *showed significant sequence similarity to the previously characterised asynapsis genes *PAIR2 *from rice, *ASY1 *from Arabidopsis and *Brassica*, and to a lesser extent, *HOP1 *from yeast. The *TaASY1 *ORF is 1764 bp, which encodes a predicted protein of 588 amino acids with a theoretical molecular weight of 66.3 kDa. Full-length protein comparisons to the other characterised asynapsis sequences revealed high levels of sequence identity (*Os*PAIR2 80%; *At*ASY1 53.8%; *Bo*ASY1 51.2% and *Sc*HOP1 16.5%). Further analysis of the translated *Ta*ASY1 sequence showed that it contained a HORMA (HOP1, REV7, MAD2) domain. The level of identity between the HORMA domains from *Ta*ASY1 compared to *Os*PAIR2, *At*ASY1, *Bo*ASY1 and *Sc*HOP1 are significant, with values of 95.7%, 78.7%, 76.3% and 24.5% respectively (Figure 1).

**Figure 2:**
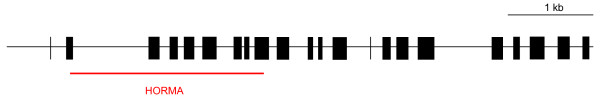
**Schematic representation of *TaASY1 *gene structure with exons (indicated by solid boxes) and introns (solid lines)**. The red line indicates the position of the HORMA domain within *TaASY1*, and the scale bar indicates a relative length of 1 Kb.

The *TaASY1 *gene structure was obtained using sequence analysis of gene specific fragments amplified via PCR from Chinese Spring. Subsequent analysis revealed that *TaASY1 *is composed of 22 exons and 21 introns and is greater than 6.5 Kb in length (Figure 2).

**Figure 3:**
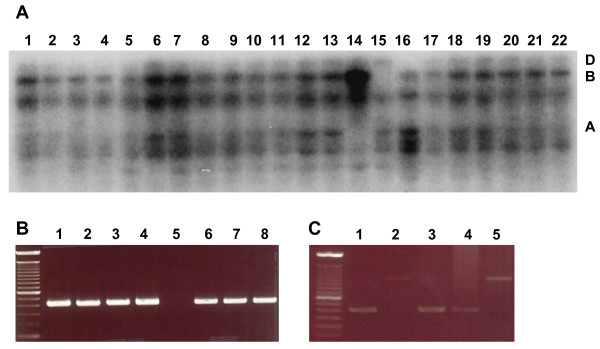
**Chromosomal location of *TaASY1 *determined by Southern blot and PCR analysis**. (A) Digested DNA from each of the 21 nullisomic-tetrasomic Chinese Spring wheat lines is shown, representing all 7 chromosome groups, which was hybridised with a full length *TaASY1 *clone labelled with α-32P dCTP. Absence of hybridisation signals in lanes 14–16, which contains DNA of lines null 5A-T5B, null 5B-T5D, null 5D-T5A respectively, illustrated that *TaASY1 *was located on chromosome group 5. (B) Amplification of the 446 bp fragment from nullisomic-tetrasomic lines with lanes 1 and 5 null 5A-T5D, lanes 2 and 6 null 5B-T5D, lanes 3 and 7 null 5D-T5A, and lanes 4 and 8 wild-type Chinese Spring. Lanes 1 to 4 contain products amplified using non-genome specific primers, and lanes 5 to 8 contain products amplified using 'A genome' specific primers, as illustrated by the lack of product in lane 5 (null 5A-T5D). (C) Amplification of the 446 bp fragment from DNA template of wild-type and various bread wheat deletion lines (lane 1 wild-type Chinese Spring, lane 2 null 5A-T5D, lane 3 mutant 5AL 4-1 (0.55 FL), lane 4 5AL 19-5 (0.35 FL) and lane 5 5AL 12-1 (0.32 FL)). The DNA molecular weight marker in (B) and (C) is a 100 bp ladder (Invitrogen, Japan).

Southern blot analysis was performed using membranes containing digested genomic DNA from the nullisomic-tetrasomic series and wild-type Chinese Spring to identify the chromosome location of *TaASY1*. Using a full length cDNA clone as a probe, *TaASY1 *was mapped to chromosomes of Group 5 (Lanes 14 to 16, Figure 3A) with a single copy of this gene on each of the three bread wheat genomes A, B and D. Interestingly, the rice orthologue of *TaASY1*, *PAIR2*, has previously been mapped to rice Chromosome 9. The long arm of rice 9 shows conservation of gene order with wheat chromosome Group 5.

We further localised *TaASY1 *using a PCR-based approach with primers specific for the A genome and multiple bread wheat deletion lines. Genome specificity of the primers was confirmed with amplification of a 446 bp fragment using template from the three nullisomic-tetrasomic lines of chromosome Group 5 and wild-type Chinese Spring (Figure 3B). Bread wheat deletion lines that contained varying distal deletions of 5A long arm (5AL) were then screened using these primers (courtesy of Professor Takashi Endo, National Bioresource Project, Kyoto University, Japan). The fragment specific for the A genome was amplified in a deletion line that contained 0.35 fraction length (FL – see Methods; DNA isolation, Southern blot and PCR analysis) of the 5AL (5AL 19-5, Professor Takashi Endo, *personal communication*), but not in a line that contained 0.32 FL of 5AL (5AL 12-1, NBRP, Kyoto University, Japan) (Figure 3C). This suggests that *TaASY1 *is located in a region between 0.32 and 0.35 FL on 5AL when measured from the centromere.

### *TaASY1 *is highly expressed in anthers at prophase I of meiosis

**Figure 4:**
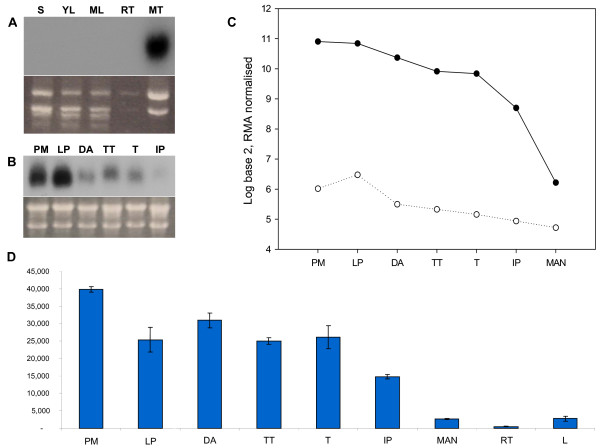
***TaASY1 *transcript expression levels observed from northern analysis, microarray and Q-PCR technology platforms**. (A) Tissue specific northern analysis of *TaASY1 *expression in seedling (S), young leaf (YL), mature leaf (ML), root tips (RT) and meiotic tissue (MT). (B) Meiosis specific northern analysis of *TaASY1 *expression during pre-meiosis (PM), leptotene to pachytene (LP), diplotene to anaphase I (DA), telophase I to telophase II (TT), tetrads (T) and immature pollen (IP). For both analyses, ethidium bromide stained rRNA was used as a loading control. (C) Using the Affymetrix GeneChip^® ^Wheat Genome Array, two probes were identified that matched *TaASY1*: Ta.9186.1.S1_at (1591 bp to 1771 bp – solid circles) and Ta.9186.2.S1_at (111 bp to 586 bp – open circles). While expression is considerably higher in Ta.9186.1.S1, it is evident that as meiosis concludes, transcript levels are minimal (as seen in both probes). PM, LP, DA, TT, T, IP as in (B) with the addition of mature anthers (MAN). (D) Tissue-specific and meiosis staged Q-PCR analysis of *TaASY1*. Expression levels detected were minimal in leaf, root and mature anthers when compared to the meiosis staged tissues investigated. PM, LP, DA, TT, T, IP, MAN as in (C) with the addition of root tips (RT) and leaf (L). Y axis is expressed as normalised mRNA copies/μL.

*TaASY1 *expression was investigated using northern analysis, microarray and quantitative real time PCR (Q-PCR) technology. In the northern blots of a tissue series (Figure 4A) and sub-staged meiotic anthers (Figure 4B), *TaASY1 *expression was found to be meiosis-specific and significantly up-regulated during pre-meiosis and leptotene to pachytene when compared to the other stages represented. Quantitative analysis of *TaASY1 *transcript levels was initially performed by microarray using the wheat Affymetrix GeneChip^®^. From the 61,127 probe sets on the wheat GeneChip^® ^which represents 55,052 transcripts, two probe sets were identical to the full length *TaASY1 *cDNA sequence that was previously isolated: Ta.9186.1.S1_at and Ta.9186.2.S1_at, at positions 1591 bp to 1771 bp and 111 bp to 586 bp, respectively. The level of expression detected for the two microarray probe sets closely reflected the northern results, with *TaASY1 *expression significantly elevated during pre-meiosis and leptotene to pachytene of prophase I (Figure 4C). Detection within the mature anthers was significantly reduced (log ratio values of 6.2 and 4.7 respectively for the two transcripts identified), indicating inactivation of *TaASY1 *transcription in this tissue. Ta.9186.1.S1_at displayed a significant decrease in transcript abundance, with a greater than 22-fold reduction between leptotene to pachytene of prophase I and the development of mature anthers.

To complement and confirm the accuracy of the microarray results, Q-PCR was also performed to investigate the transcript expression levels of *TaASY1 *across a range of tissues. While low levels of expression were evident in leaf and root tips which were not detected in the tissue series northern, the results revealed significant levels of expression in anthers during the early stages of meiosis (Figure 4D), confirming the results obtained from the other two techniques used. In parallel, the microarray and Q-PCR platforms exhibited a correlation value of 0.98 between each other thus suggesting that the results were highly reproducible [30].

These assays all used RNA isolated from whole wheat anthers and contained many tissues in addition to the meiocytes; including tapetum, epidermis, endothecium and segments of the filament. Demonstration of meiocyte expression and a role for the *Ta*ASY1 protein in pairing required sub-cellular localisation of the gene product.

### Protein analysis validates *TaASY1 *expression in meiotic tissue and location adjacent to the axial elements of chromosomes at early prophase I

**Figure 5:**
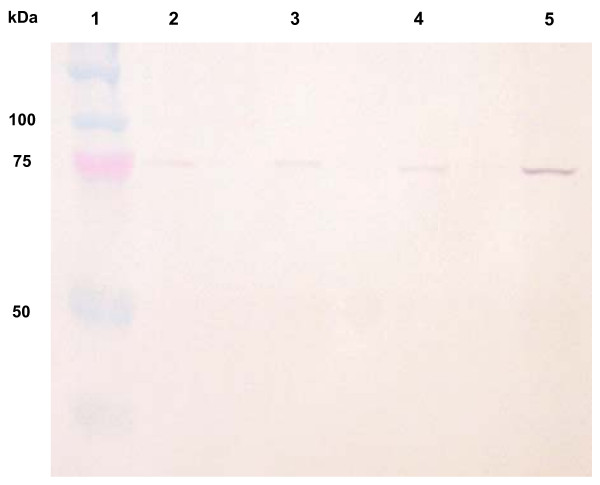
**Western blot analysis using anti-*Ta*ASY1 immune sera**. Protein was detected in young leaf (lane 2), mature leaf (lane 3), root (lane 4) and meiotic tissue (lane 5). The molecular weight marker is highlighted in lane 1 (Precision Plus Dual Color, Bio-Rad, CA, USA). The detection of protein is significantly elevated in the meiotic tissue lane when compared to the vegetative tissues examined.

Using a *Ta*ASY1 specific antibody, a high level of protein was detected in meiotic tissue (Figure 5), as expected from the transcript analyses. This protein was approximately 70 to 75 kDa in size. The antibody also detected a faint signal in the protein samples of each vegetative tissue analysed (young leaf, mature leaf and root).

**Figure 6:**
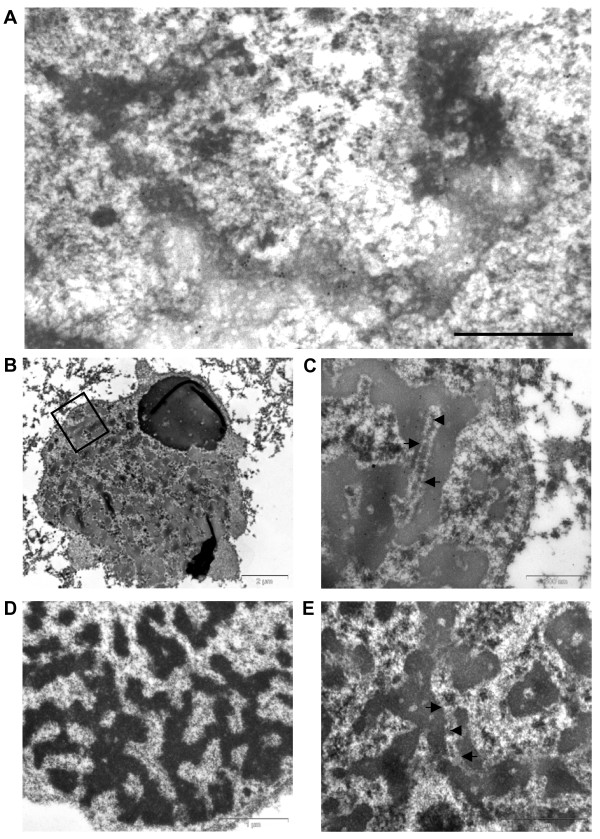
**Immunogold localisation of *Ta*ASY1 polyclonal antibody using anther sections from *T. aestivum***. (A) Immunogold labelling of meiotic cells at early zygotene showing localisation of *Ta*ASY1 to dense axial elements (bar = 0.5 μm) (B) Low magnification image of a meiotic cell at late zygotene to early pachytene, containing formed SCs, as illustrated by the SC within the boxed area, which is shown in (C). (C) Immunogold labelling displays localisation of *Ta*ASY1 to chromatin regions associated with the lateral elements. Chromosome axes (lateral elements) are indicated with arrows, and the central element is indicated with an arrow head. (D) Diplotene meiotic cell showing loss of *Ta*ASY1 localisation once the SC has disassociated and chromosomes have condensed further. (E) Negative control excluding the primary antibody. Arrows indicate chromosome axes and the arrowhead indicates the central element. All sections were counterstained with uranyl acetate and lead citrate.

Analysis by transmission electron microscopy (TEM), using sections of anthers that contained cells undergoing meiosis, revealed that the *Ta*ASY1 antibody specifically labelled the nucleus and structures that form part of the SC. During leptotene and early zygotene, *Ta*ASY1 associates with chromatin adjoining axial elements (Figure 6A) similar to that seen with *At*ASY1 and *Os*PAIR2 [24,25]. Upon complete SC formation in pachytene nuclei, *Ta*ASY1 was found to interact with axis-associated chromatin, with labelling present amongst dense chromatin adjacent to lateral elements of the tri-partite SC (Figure 6B, C) [see Additional file 1]. Labelling was not observed in nuclei of cells that were progressing through diplotene and diakinesis, suggesting that the protein is degraded or removed upon disassociation of the SC (Figure 6D). The two negative controls including grids without primary antibody (Figure 6E) and grids labelled with a goat anti-rabbit secondary antibody (data not shown) displayed no labelling.

## Discussion

Primarily, chromosome pairing and synapsis in bread wheat has been extensively analysed using mutants, such as *ph1b *and *ph2a*, which display reduced restriction of chromosome pairing to homologues (reviewed in [31,32]). The major loci (*Ph1 *and *Ph2*) responsible for this phenotype have been mapped to 5BL and 3DS, respectively. Since their discovery many cytological studies have been conducted to investigate the diploid behaviour of chromosome pairing during meiosis in bread wheat [1,2,7,33-40]. More recently, significant work on chromosome pairing dynamics has led to theoretical models which help explain how this complex organism maintains its diploid behaviour during meiosis [1,6,7,32]. Coupled with the cytological studies have been the extensive mapping strategies of Griffiths and colleagues [41] to identify the gene(s) responsible for the *Ph1 *phenotype, culminating in the identification of four *cdc2*-related genes and a sub-telomeric heterochromatic region that translocated from chromosome 3AL, as candidates for the *Ph1 *effect. Theses approaches have been built around the use of the genetic information to track down genes that control pairing. There have been few reports of studies aimed at deciphering components of the wheat meiotic machinery based on candidates identified in other systems. The characterisation of *TaASY1 *described here suggests that knowledge from other systems can be applied to bread wheat and can help build a molecular view of pairing and recombination.

While the presence of *TaASY1 *on chromosome Group 5 implies that it does not represent *Ph2*, its location and putative role, based on the asynaptic phenotype of Arabidopsis and rice mutants, suggests it may still be involved with either the product of the *Ph1 *and/or *Ph2 *loci; albeit indirectly. Based on the findings of Griffiths et al. [41], and Southern analysis that we have conducted using the *Ph1 *mutant, *ph1b *(data not shown), *TaASY1 *does not represent *Ph1*. Given its location on the long arm of chromosome 5A, it is possible that *TaASY1 *represents one of the previously reported minor chromosome pairing promoters on 5AL and/or 5DL [36,42,43]. However, further experimentation will be required to determine if any of these loci represent *TaASY1*. This work is likely to involve precise location of the gene relative to various bread wheat deletion lines that have previously been reported [36,42-44].

*TaASY1*, *ASY1 *from Arabidopsis and *Brassica*, and *PAIR2 *from rice represent the only HORMA domain containing proteins identified in plants [19,20,24]. The amino acid sequences of the ASY1 and PAIR2 proteins display significant sequence similarity to *Ta*ASY1. When comparing only the HORMA domain of all known asynaptic proteins reported, sequence identity increased significantly suggesting that the HORMA domain is essential for the function of *Ta*ASY1 and its orthologues. However, this is not always the case when comparing sequences of meiotic proteins across various organisms, with sequence identities varying widely for proteins involved in the evolutionarily conserved process of chromosome pairing and synapsis [12,14-16,45,46]. This is highlighted by the difficulty to predict meiotic activity of *Os*PAIR2 based around the extensive knowledge of *Sc*HOP1 [25,45,47-53].

The *TaASY1 *gene structure is very similar to the reported structures of *AtASY1 *and *OsPAIR2*, with all three genes having 22 exons and 21 introns. The similarity in gene structure of *TaASY1 *and *OsPAIR2 *is also reflected in the location of the two genes, with chromosome Group 5 of bread wheat displaying high levels of gene order conservation with rice Chromosome 9, on which *OsPAIR2 *resides [20].

The three transcript analysis procedures used in this study clearly indicated significant expression of *TaASY1 *in meiotic tissue, with highest expression predominantly confined to pre-meiosis and leptotene to pachytene. The leptotene to pachytene result was expected based on these stages being when synapsis occurs, which is also reflected by the phenotypes of *asy1 *and *pair2 *mutants where chromosomes fail to synapse [18,20].

The Q-PCR data indicated that transcript levels of *TaASY1 *remained elevated from diplotene through to the completion of telophase II. This may be due to minor asynchrony between the collection of anthers that were selected for staging and subsequent RNA isolation. In addition, as wheat meiosis takes only 24 hours to complete it is possible that the elevated expression seen later in meiosis represents the mRNA that remained in the cells as they rapidly progressed through the meiotic cycle, after the relatively lengthy prophase I period of 17 hours [54]. Although the *TaASY1 *transcript was not detected in vegetative tissues using northern analysis, Q-PCR and microarray analysis suggested that there were very low levels of expression (up to approximately 40,000 fold less). Both these technology platforms are extremely sensitive to very low transcript levels.

The elevated transcript expression in meiotic tissue correlates with the detection of protein seen in the western analysis. The very low level of protein in the leaf and root tissues contrasts to results from Arabidopsis and rice [24,25]. However, the differences detected between the reports could be attributed to alternative experimental procedures used, including the protein extraction procedure and the higher concentration of primary antibody used in this study.

While it was not possible to define whether *Ta*ASY1 associated directly with axial elements, immunolocalisation using TEM revealed that *Ta*ASY1 associates with chromatin regions of axial elements prior to formation of the SC, as well as chromatin of lateral elements within a formed SC. These results are consistent with the previous data in Arabidopsis and rice where ASY1/PAIR2 are shown to have a role in pairing and synapsis of homologous chromosomes. Although *Ta*ASY1 is located adjacent to axial elements prior to SC formation, it is unlikely to have a role in initiation of axial element formation since dense axial elements still formed in the rice *pair2 *mutants. In addition, as the axial elements that formed in the *pair2 *mutant were comparable in size to those in the wild-type plant, especially when compared to the differences in axial element size and distribution of ASY1 in the maize mutant *afd1*, it is unlikely that the labelling of *Ta*ASY1 to axial elements in wheat is due to a role in axial element elongation [25,26].

Localisation of *Ta*ASY1 to components of the SC both before and after SC formation indicates a role for this protein in synapsis and/or pairing of homologous chromosomes. A role for *Ta*ASY1 in synapsis is supported by results of Mikhailova et al. [27]. Using a rye mutant that failed to correctly synapse homologous chromosomes, Mikhailova et al. [27] demonstrated that ASY1 and ZYP1 (a known SC component) still load correctly onto chromosome axes without formation of the tripartite SC. It is plausible therefore that the localisation of *Ta*ASY1 to axial elements prior to synapsis may represent a role in recruiting components of the SC, such as ZYP1, to the axial elements in preparation for SC formation.

While support for a role in chromosome pairing is less obvious, it has been shown that the Arabidopsis *asy1 *mutant exhibits near normal centromere and telomere pairing behaviour during interphase and leptotene but subsequent stages are atypical, with non-recognisable pairing and synapsis of homologues [55]. This indicates that ASY1 is active between the time point where telomeres and centromeres first associate and homologues correctly synapse. Therefore, in bread wheat *TaASY1 *represents an interesting candidate for further research into how homologues are resolved from their homoeologues, since the seven homoeologous centromere clusters form prior to the resolution of 21 homologous chromosome pairs [7].

A long term objective of this research is to pursue an efficient methodology for the induction of pairing control and recombination in bread wheat. Significant progress was recently made towards this goal with the molecular characterisation of the *Ph1 *locus by Griffiths et al. [41]. However, to understand the mechanism of action of *cdc2*-kinases found at the *Ph1 *locus, it may be necessary to characterise down stream proteins involved in chromosome synapsis, such as *Ta*ASY1. To this end we have begun investigating what proteins interact with *Ta*ASY1 and where these proteins are located within the bread wheat genome.

## Conclusion

We have isolated and characterised the wheat homologue of *ScHOP1*, *AtASY1 *and *OsPAIR2*; called *TaASY1*. This study has enhanced our understanding of proteins that are responsible for the correct pairing and synapsis of homologous chromosomes in bread wheat. *TaASY1 *is located on Chromosome group 5, with a copy on each of the three genomes; A, B and D. Transcript and protein expression analyses indicate a role for this protein during the early stages of meiosis, specifically during prophase I. This was confirmed by immunolocalisation using TEM, which showed that *Ta*ASY1 interacts with chromatin of SC associated structures during zygotene and pachytene, before being removed or degraded during later stages.

## Methods

### Plant materials

Hexaploid wheat plants including wild-type (*Triticum aestivum *cv. Chinese Spring), nullisomic-tetrasomic (NT) derivatives, and mutants of wild-type Chinese Spring (*ph2a*, *ph2b*, *ph1b*) and a series of 5AL deletion lines (courtesy of Professor Takashi Endo, NBRP, Kyoto University, Japan) were grown under a 14 hour photoperiod in a temperature controlled glasshouse ranging from 15°C to 23°C.

### DNA isolation, Southern blot and PCR analysis

Plant genomic DNA extraction and Southern blot analysis was conducted according to [56]. The *TaASY1 *full-length ORF cDNA was amplified and used as a probe, from the cultivar Chinese Spring using primers *TaASY1*F1 (5' ATGGTGATGGCTCAGAAGACG) and *TaASY1*R1 (5' TGAACTAGGACTTCTGGCGC).

The probe was labelled using α-32P dCTP and hybridised to membranes according to [57]. PCR analysis of Chinese Spring mutants containing varying deletions of chromosome 5AL was performed to identify the location of *TaASY1*. Genome specific primers were designed for the A genome based on genomic DNA sequence comparisons of *TaASY1 *amplified from the three nullisomic-tetrasomic lines of chromosome group 5 (TGS1AS; 5' CCACGCTCATCTTGTCATCATCA 3', TGS2S; 5' GTTATCGACAGCTGCCATCCTAGA 3'). The eight mutants analysed were: n5AL.4-1, n5AL.12-1, n5AL14-1, n5AL14-3, n5AL14-4, n5AL14-5 and n5AL.19-5. Each mutant contained varying deletions (fraction length – FL) of 5AL, as detailed on the National BioResources Project database (courtesy of Professor Takashi Endo, Kyoto University) [58].

### Meiotic staged tissue and whole plant tissue collections

Meiotic tissue from the plant material listed was harvested early in the morning. With the complete inflorescence having been dissected from the sheath, individual florets from the central region of the spike were prepared for anther squashes. Using aceto-orcein to stain the meiocytes, compound light microscopy was used in order to determine the stage of meiosis. Upon determining the stage, the remaining anthers from the corresponding floret were placed in a microfuge tube in liquid nitrogen. This process was repeated for florets up and down the length of several wheat inflorescences, with anthers from identical stages being pooled.

Anthers from the following stages were collected: pre-meiosis, leptotene to pachytene, diplotene to anaphase I, telophase I to telophase II, tetrads, immature pollen and mature pollen. In addition to the meiosis specific sub-staged tissue collection, complete immature inflorescences, mature leaf, young leaf, roots and seedlings were also collected. This process was conducted twice; once for the northern analysis and the second time for both microarray and Q-PCR analysis.

### RNA isolation and northern blot analysis

RNA was extracted from the following: whole seedling (14 days), young leaf (21 days), mature leaf (56 days), root tissues (14 days) and immature inflorescence material. In addition, staged meiotic anthers were also collected for RNA extraction from pre-meiosis, leptotene to pachytene, diplotene to anaphase I, telophase I to telophase II, tetrads and immature pollen. RNA was extracted using Trizol reagent (Gibco BRL, Australia) according to manufacturer's instructions.

For northern analysis, 5 μg of total RNA for each sample was separated on a 1% denaturing agarose gel and subsequently transferred to Hybond N^+ ^membrane (Amersham Biosciences, Australia). RNA loading equivalents were visualised prior to membrane transfer, using the GeneFlash gel documentation system (Syngene BioImaging, USA). Hybridisation was conducted in formamide solution at 42°C [59]. Membranes were washed and film developed as in Sutton et al. [57].

### cDNA synthesis

Prior to cDNA synthesis, the total RNA samples were first treated with DNase I using the TURBO-DNA free kit, according to manufacturer's instructions (Ambion, Australia). Synthesis of cDNA was performed using SuperScript III (Invitrogen, Australia), according to the manufacturer's instructions.

### Isolation of *TaASY1 *using inverse PCR

An EST sequence (Accession Number: CA599825) that was identified as having significant similarity to *AtASY1 *(Accession Number: AF157556) was used as the basis for *TaASY1 *isolation. To isolate the full length *TaASY1 *cDNA, an inverse PCR method was utilised. Total RNA was extracted from immature inflorescences using Trizol reagent (Gibco BRL, Australia) according to manufacturer's instructions. 5 μg of total RNA was reverse transcribed using oligo dT_(12–18) _and the Superscript III kit (Invitrogen, Australia) as per manufacturer's instructions. Following phenol chloroform purification of first strand cDNA, the 3' ends were tailed with dATP using terminal transferase (Invitrogen, Australia) as per manufacturer's instructions. The poly A capped single stranded cDNA was ethanol precipitated and half the volume was used for second strand cDNA synthesis (50°C, 2 minutes; 72°C, 20 minutes; 30 cycles of 94°C, 1 minute; 50°C, 2 minutes; 72°C, 10 minutes; with a final elongation at 72°C for 10 minutes) using primer *B26 *5' GACTCGAGTCGACATCGAdT_(17) _and Q *Taq *(QIAGEN, Australia).

The double stranded cDNA was ethanol precipitated and 100 ng was used in a 150 μL ligation reaction using T4 DNA ligase (New England Biolabs, Australia) as per manufacturer's instructions. Circularised double stranded cDNA was then heat denatured, phenol chloroform purified and resuspended in 10 μL of water. Primers *Asyrev2 *(5' TCATCTGGTCAGGAGTGACTTCTGCTG) and *Asyfwd1 *(5' GCAAAGGTCAGAGTGGTACAAACTC) were used to amplify the full length *TaASY1 *clone from 1 μL of the circularised double stranded cDNA using High Fidelity *Taq *Polymerase (Roche, Australia) as per manufacturer's instructions with the following cycling parameters: 94°C, 1 minute; 35 cycles of 94°C, 30 seconds; 55°C, 30 seconds; 68°C, 3 minutes; with a final extension of 68°C for 10 minutes. The *TaASY1 *product was cloned into pGEM T-easy (Promega, Australia) and sequenced as described below. The final cDNA clone obtained was 2145 bp.

### Genomic clone isolation and sequencing

Isolation of the *TaASY1 *genomic DNA sequence was obtained by PCR using Chinese Spring genomic DNA as template and a number of primer sets, as outlined in Table 1. The fragments obtained were cloned into pGEM T-easy (Promega, Australia) and sequenced as described below.

**Table 1:** Primer sets used to determine the gene structure of *TaASY1*

**Region**	**Sense Sequence**	**Antisense Sequence**	**Product size**
Exon 1–3	CCACGCGCGCACACAACACA	GCAATCCGGAGCAAATTCCT	2.2 Kb
Exon 2–3	GCTTGCTTGCTCGGTTCCATTTG	CCAACCAATAATCCTGCAATGTATTTG	1.0 Kb
Exon 3–9	GGTGTCTATGATGCCTTACAAAAG	CTTGCTATTGACATTCCCCAC	1.6 Kb
Exon 10-Intron 14	GCTAATGATGCTAACAGTGATGATGAC	GTGGACTAACACTATAAAGAAATCTG	0.8 Kb
Exon 7–17	CGCTAGTTTCACTTATGAGGACCTTG	CCATCAAGCTTGCCCTGAAG	1.9 Kb
Exon 16-Intron 20	GGAAGAGGTTGCCATGCACAAT	CCAAACCCAATGTGACAGAGGTAAG	1.2 Kb
Exon 16–22	GGAAGAGGTTGCCATGCACAAT	GTTGCGCCGAGGCTCCTTGC	1.8 Kb

### Affymetrix wheat GeneChip^® ^microarray hybridisation and expression analysis

For experimental procedures regarding the microarray hybridisation and expression analysis, refer to Crismani et al. [30].

### Q-PCR expression analysis

Q-PCR was conducted as per [60] with the modifications and *TaASY1 *specific primers that are reported in [30].

### Sequence analysis

Nucleotide sequencing was conducted using the BigDye™ Terminator Sequencing v3.1 Ready Reaction Kit (Perkin Elmer, USA). Sequence PCR products were cleaned for analysis using 75% isopropanol, prior to sequencing using an ABI Prism 3700 DNA Analyser (Applied Biosystems) at the Institute of Medical and Veterinary Science (IMVS, Adelaide, Australia). Sequence data was analysed using VNTI Suite Version 8.0 software (Informax Inc., MD, USA). To assign putative functions to sequenced products, BLASTn, tBLASTn, tBLASTx, BLASTp searches of the GenBank non-redundant databases were conducted.

### Antibody production

A peptide spanning residues 486 to 499 (DRRDHQTADQEMKDC) of the *Ta*ASY1 amino acid sequence was synthesized (AusPep, Australia). Selection of the peptide was based on its low hydro-phobicity, uniqueness of sequence when used in a BLAST search against known translated sequences and protein sequences (tBLASTn and BLASTp), and its predicted structure compared to protein structures with sequence similarity to *Ta*ASY1. The peptide was initially dissolved in 200 μL of 1× PBS (10 μg μL^-1^) and conjugated with an equal volume of the carrier molecule KLH (Keyhole Limpet Hemocyanin, Pierce, Australia) dissolved in double autoclaved milli-Q water (10 μg μL^-1^). For the first mouse immunization, 50 μL ofKLH-conjugated antigen was added to 50 μL of 1× PBS, and subsequently added to an equal volume of Freund's complete adjuvant (Sigma-Aldrich, Australia) prior to subcutaneous injection. For the following immunisations, which were administered at three weekly intervals, the 100 μL ofKLH-conjugated antigen in 1× PBS was added to Freund's incomplete adjuvant (Sigma-Aldrich, Australia) prior to subcutaneous injection. Immune sera were extracted 68 days after the first injection.

### Protein production

To generate recombinant *Ta*ASY1 protein, the full length *TaASY1 *ORF was inserted into pCR8/GW/TOPO (Invitrogen, Australia). The full length clone was inserted into the pDEST17 vector containing a 6× histidine (6× His) repeat at the 5' end of the entry site using Gateway technology, according to manufacturer's instructions (Invitrogen, Australia). Protein production in BL21-A1 cells (Invitrogen, Australia) was induced by adding L-arabinose (Sigma-Aldrich, Australia) to a final concentration of 0.2% in LB liquid culture. The recombinant protein was then extracted and purified using Ni-NTA agarose under denaturing conditions according to manufacturer's instructions (QIAGEN, Australia). Trypsin digestion mediated mass spectrometry using a QTOF^2 ^mass spectrometer confirmed that the recombinant protein expressed was *Ta*ASY1 [see Additional file 2]. The resulting MSMS were analysed using the ProteinLynx Global Server to search against the NCBI non-redundant database for peptide tag matches. This was also combined with *de novo *sequencing and BLAST analysis.

### Western blot analysis

Proteins were extracted from plant tissue using a phenol extraction and methanol ammonium acetate precipitation method [61]. Protein samples were quantified using the Bradford method [62], and equal amounts of protein (10 μg) from each tissue sample were loaded on a 7.5% polyacrylamide gel for separation by SDS-PAGE. Protein samples were then electroblotted onto a Hybond-P polyvinylidene difluoride (PVDF) membrane (Amersham Biosciences, Australia).

Western blots were incubated with anti-*Ta*ASY1 antiserum diluted 1/1500 followed by incubation with anti-mouse IgG antibodies conjugated to biotin (Sigma-Aldrich, Australia) diluted 1/1000. Streptavidin conjugated to alkaline phosphatase (Sigma-Aldrich, Australia) was then added at a dilution of 1/1000 followed by addition of BCIP/NBT Purple Liquid Substrate System (Sigma-Aldrich, Australia) for protein detection.

### Immunolocalisation

Chinese Spring anthers were collected, stained and prepared as described earlier to determine their meiosis stage. Partner anthers of those determined via staining to be of the prophase I sub-stages leptotene through to pachytene were incubated in fixative solution (0.25% gluteraldehyde, 3% paraformaldehye, 4% sucrose in 1× PBS) overnight at 4°C. Fixed anthers were then washed three times with 1× PBS for 8 hours each, before being dehydrated in an ethanol series of 70%, 90%, 95% and 100% consecutively, with three lots of 20 minute incubations in each solution. Anthers were then incubated in a 50:50 mixture of 100% ethanol and LR white resin for 8 hours, before being incubated three times in pure LR white resin for 8 hours each. Anthers were then embedded into LR White Resin by incubation at 60°C for 60 hours. Standard procedures using an ultramicrotome were then used to produce tissue sections for TEM, which were affixed to nickel mesh grids.

Sectioned grids were then prepared for immunolocalisation by incubating with 0.05 M glycine for 20 minutes, followed by 2 incubations with incubation buffer (1 × PBS/0.15% AURION BSA-c™). Sections were then incubated for 90 minutes with anti-*Ta*ASY1 mouse polyclonal antiserum diluted 1/400 in incubation buffer. The sections were then washed 3 times with incubation buffer, before being incubated with a gold conjugated goat anti-mouse IgG serum (Aurion, Wageningen, The Netherlands) diluted 1/30 with incubation buffer. This was followed with three washes using both incubation buffer and 1 × PBS, with fixation of sections using 2% glutaraldehyde in PBS. Samples were then washed two times in both PBS and distilled water. Prior to visualisation, the grids were counter-stained with uranyl acetate (10 minutes) and lead citrate (5 minutes), with three 1 minute washes in distilled water after each stain. Stained and labelled sections were visualised using a Philips 100 transmission electron microscope, with images recorded using a SIS Megaview II CCD camera and AnalySIS software (Soft Imaging Systems) at Adelaide Microscopy (Adelaide, Australia).

### Accession number

The *TaASY1 *sequence (accession: EF446137) has been deposited with NCBI.

## Authors' contributions

SAB and JAA designed and conducted the research, analysed the data and drafted the manuscript. NS produced the antibody. ET conducted the northerns, analyzed the data and drafted the manuscript. PL analysed the data and drafted the manuscript. All authors approved the final manuscript.

## Supplementary Material

Additional file 1 Immunolocalisation using transmission electron microscopy (TEM). Additional TEM images that support Figure 6 within the manuscript and a schematic showing the immunolabelling are presented. Click here for file

Additional file 2 QTOF^2 ^mass spectrometry data for *Ta*ASY1. Mass spectrometry report for the wheat ASY1 recombinant protein. Click here for file
