# Supplementary material for: Expression and functional analysis of TaASY1 during meiosis of bread wheat (Triticum aestivum)
Source: BMC Mol Biol. 2007 Aug 4;8:65. doi: 10.1186/1471-2199-8-65 (PMC1971066; doi:10.1186/1471-2199-8-65)
Supplement: Additional file 1 — Immunolocalisation using transmission electron microscopy (TEM). Additional TEM images that support Figure 6 within the manuscript and a schematic showing the immunolabelling are presented. [file 1471-2199-8-65-S1.ppt]

## Slide 1
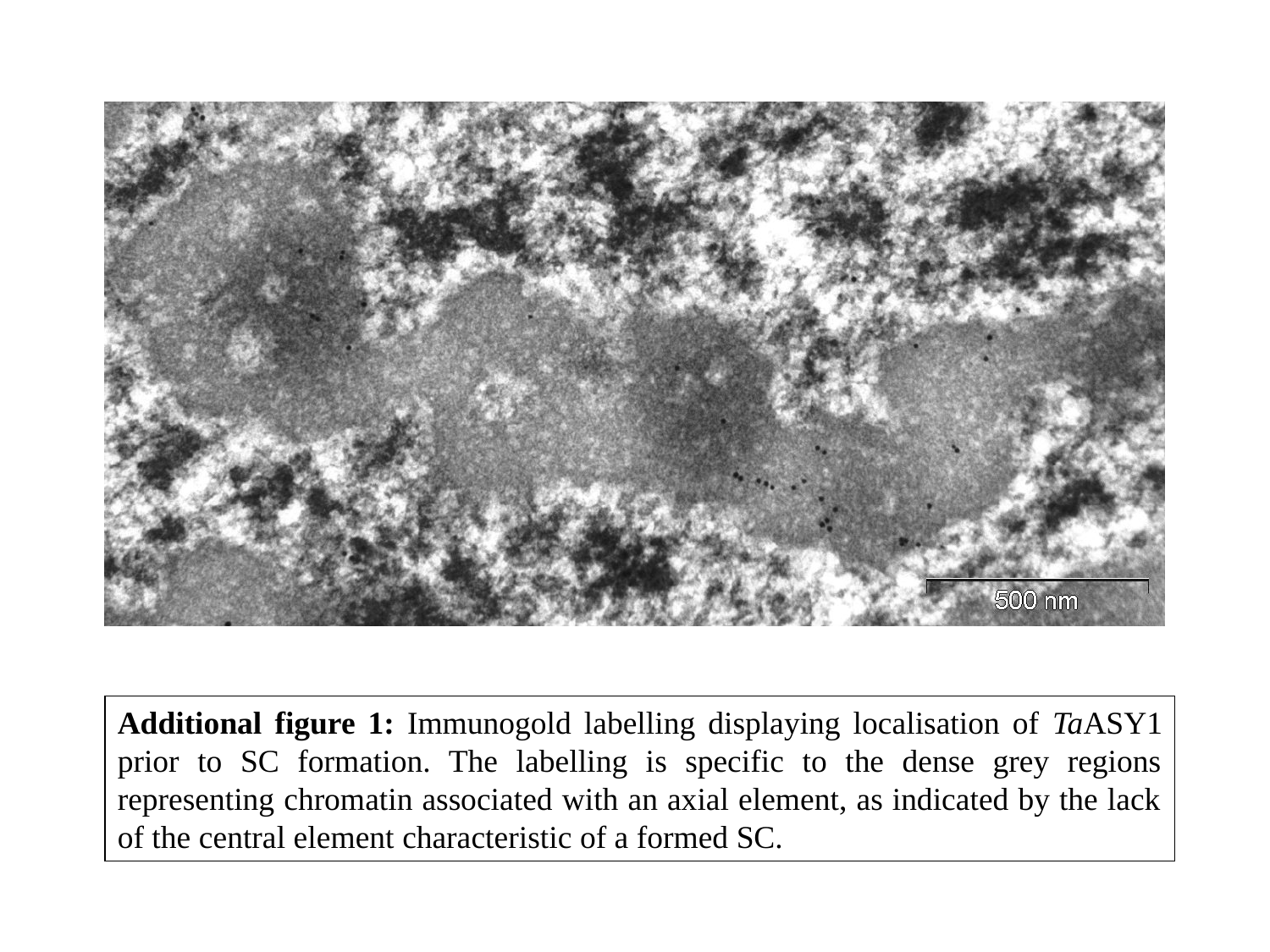

Additional figure 1: Immunogold labelling displaying localisation of TaASY1 prior to SC formation. The labelling is specific to the dense grey regions representing chromatin associated with an axial element, as indicated by the lack of the central element characteristic of a formed SC.

## Slide 2
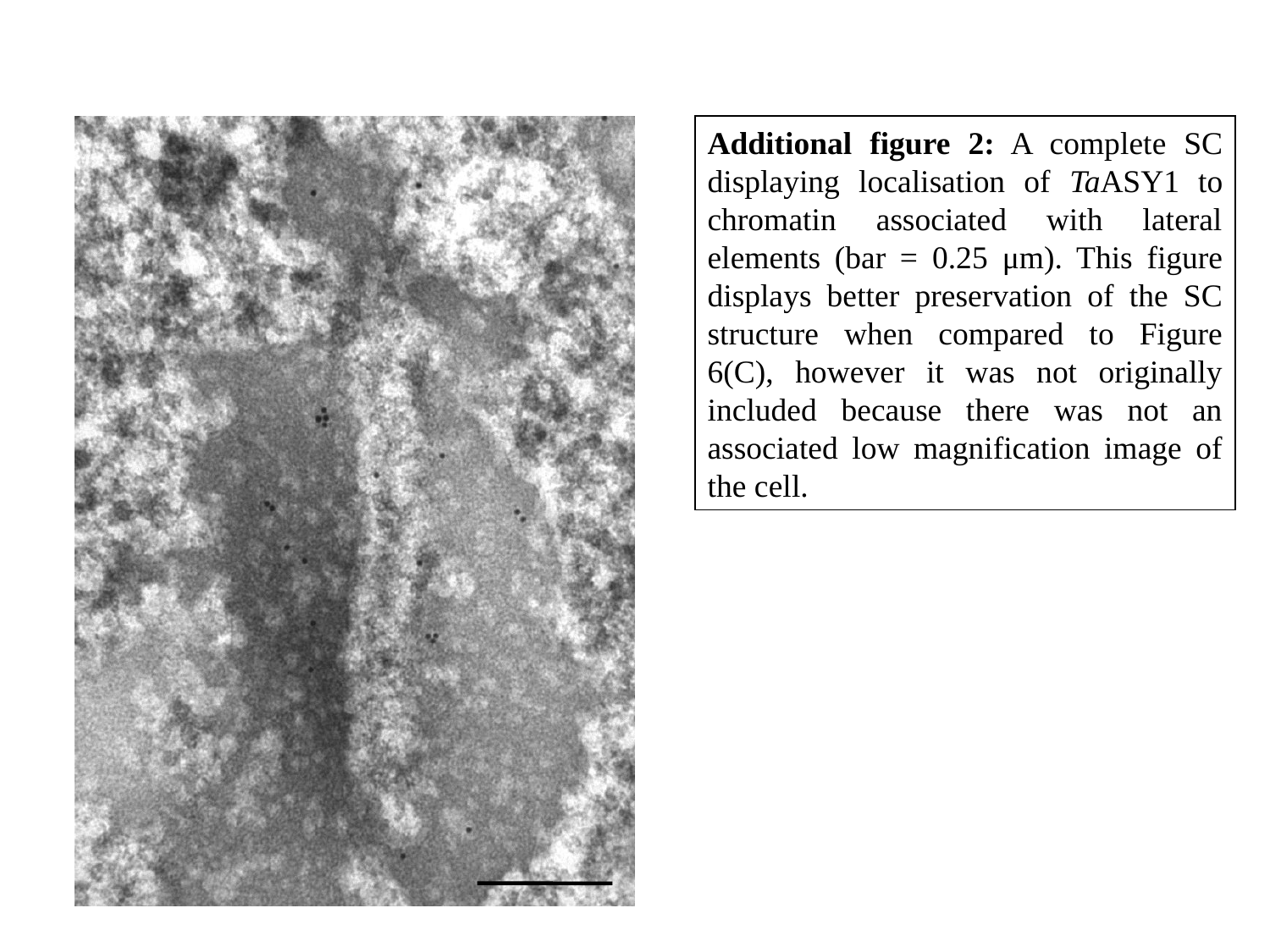

Additional figure 2: A complete SC displaying localisation of TaASY1 to chromatin associated with lateral elements (bar = 0.25 μm). This figure displays better preservation of the SC structure when compared to Figure 6(C), however it was not originally included because there was not an associated low magnification image of the cell.

## Slide 3
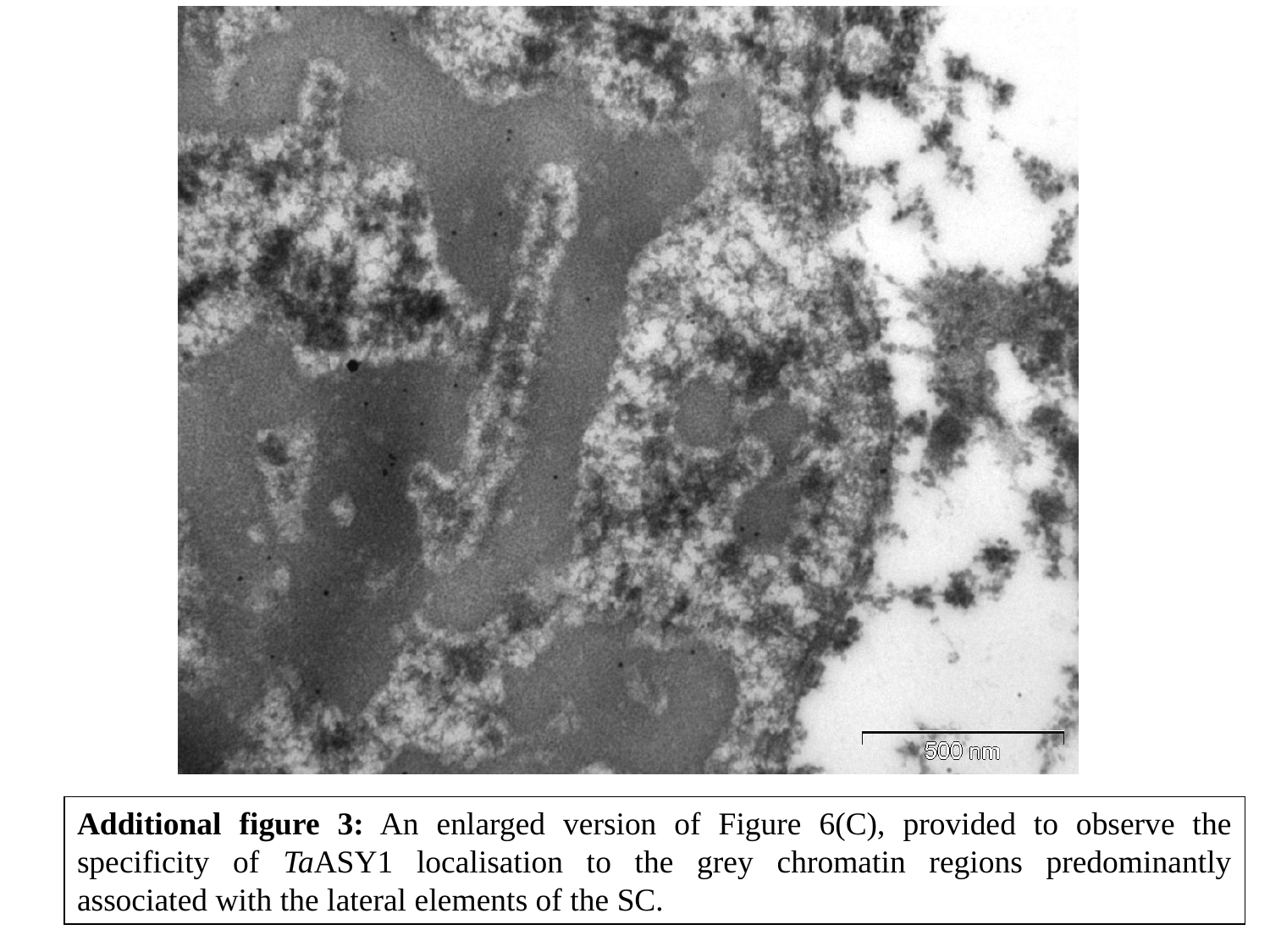

Additional figure 3: An enlarged version of Figure 6(C), provided to observe the specificity of TaASY1 localisation to the grey chromatin regions predominantly associated with the lateral elements of the SC.

## Slide 4
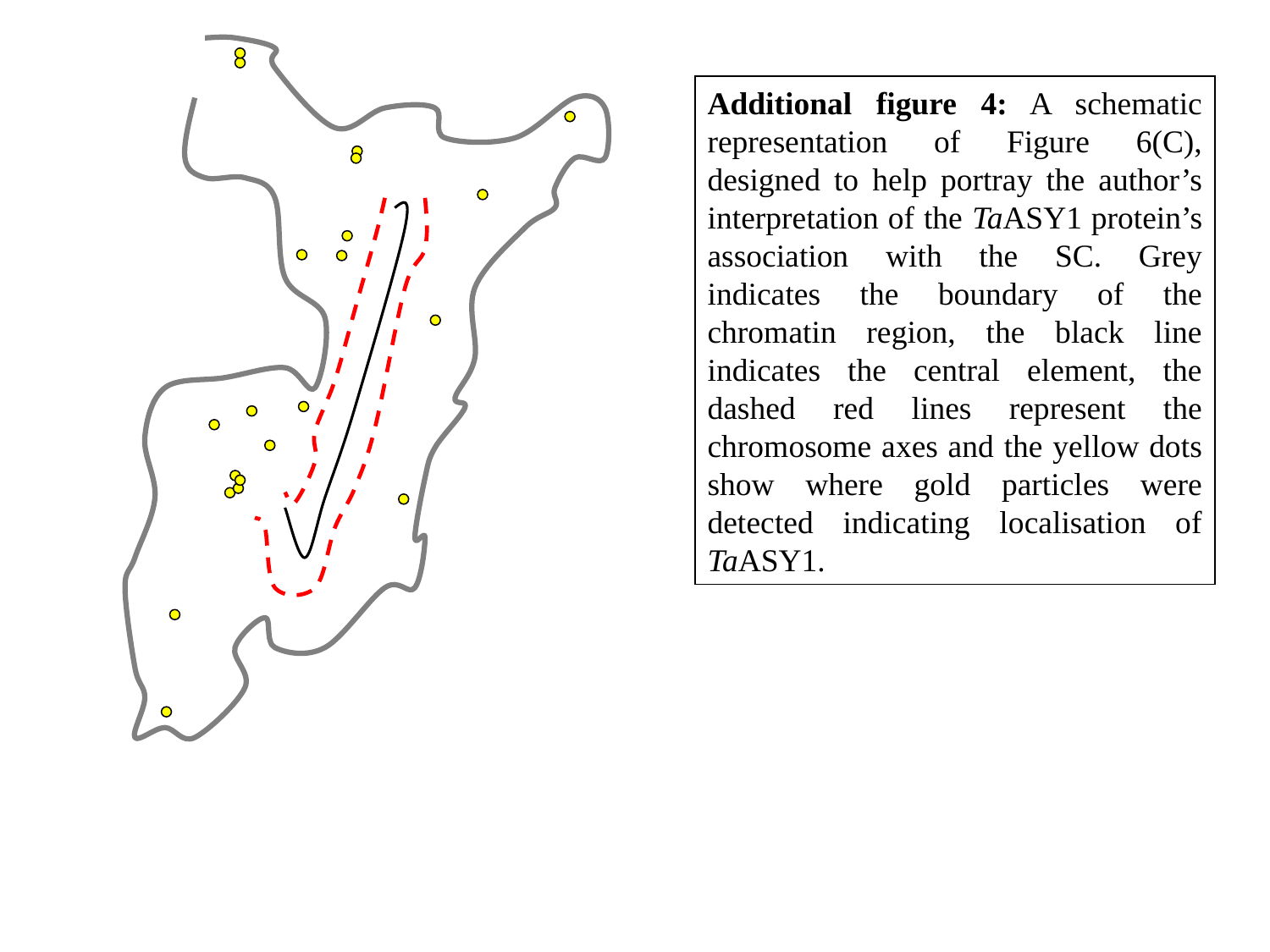

Additional figure 4: A schematic representation of Figure 6(C), designed to help portray the author’s interpretation of the TaASY1 protein’s association with the SC. Grey indicates the boundary of the chromatin region, the black line indicates the central element, the dashed red lines represent the chromosome axes and the yellow dots show where gold particles were detected indicating localisation of TaASY1.
