# Supplementary material for: Expression and functional analysis of TaASY1 during meiosis of bread wheat (Triticum aestivum)
Source: BMC Mol Biol. 2007 Aug 4;8:65. doi: 10.1186/1471-2199-8-65 (PMC1971066; doi:10.1186/1471-2199-8-65)
Supplement: Additional file 2 — QTOF2 mass spectrometry data for TaASY1. Mass spectrometry report for the wheat ASY1 recombinant protein. [file 1471-2199-8-65-S2.pdf]

Mass Spectrometry Report

Jan 20, 2006

Hanson Institute
Protein Core Facility

Sample: C:\MS Files\20-1-06\06-006-1.raw

Samples for identification by mass spectrometry were digested with trypsin under standardised conditions. The resulting peptides were reduced with TCEP and desalted through a C18 reverse phase silica column into the Q-Tof2 Mass Spectrometer, via a NanoSource.

The spectrometer was calibrated against the fragmentation pattern of [Glu]-Fibrino peptide B and found to be accurate to within 30 ppm.

Data was collected as intensity versus mass over charge (Th, Thompsons) and multiply charged ions (+2, +3, +4) were automatically detected and subjected to fragmentation.

The collected fragmentation data was the analysed using ProteinLynx software and searched against a FASTA protein database.

Protein identification matches were assigned if 2 or more sequenced peptides were identified from a protein in the database.

Regards,

Hanson Institute Protein Core Facility
Division of Human Immunology Level 3
IMVS Building Frome Road, Adelaide,
SA, 5000

Workflow Template: *Medium NCBI Search*

Property	*Value*
Query Type	*Databank Search*
Search Type	*PLGS*
Databank	*NCBI0505-1.0*
Fragment Tolerance	*0.1 Da*
High MW Filter	*200000*
Low MW Filter	*0*
High Pi Filter	*14.0*
Low Pi Filter	*0.0*
Mass Tolerance	*100.0Da*
Min Peptides	*2*
Min Std. Deviation	*0.0050*
Digestor 1	*Trypsin*
Query Type	*Automod Search*
Consider Mods	*true*
Consider Subs.	*true*
Max. Mods & Subs	*1*
Fragment Tolerance	*0.1*
Mass Tolerance	*50.0 Da*
Min. Mass Std. Deviation	*0.0050*
Digestor 1	*Trypsin*

Sample: C:\MS Files\20-1-06\06-006-1.raw

Acc #	Protein Description	Peptides	Coverage	Score
37999050	essential protein for meiotic synapsis Oryza sativa japonica cultivar ...	5	7.377	14.7098

Dr Chris Bagley chris.bagley@imvs.sa.gov.au (08) 8222 3714

Dr Ian Milne ian.milne@imvs.sa.gov.au (08) 8222 3716

MS Spectrum   C:\MS Files\20-1-06\06-006-1.raw

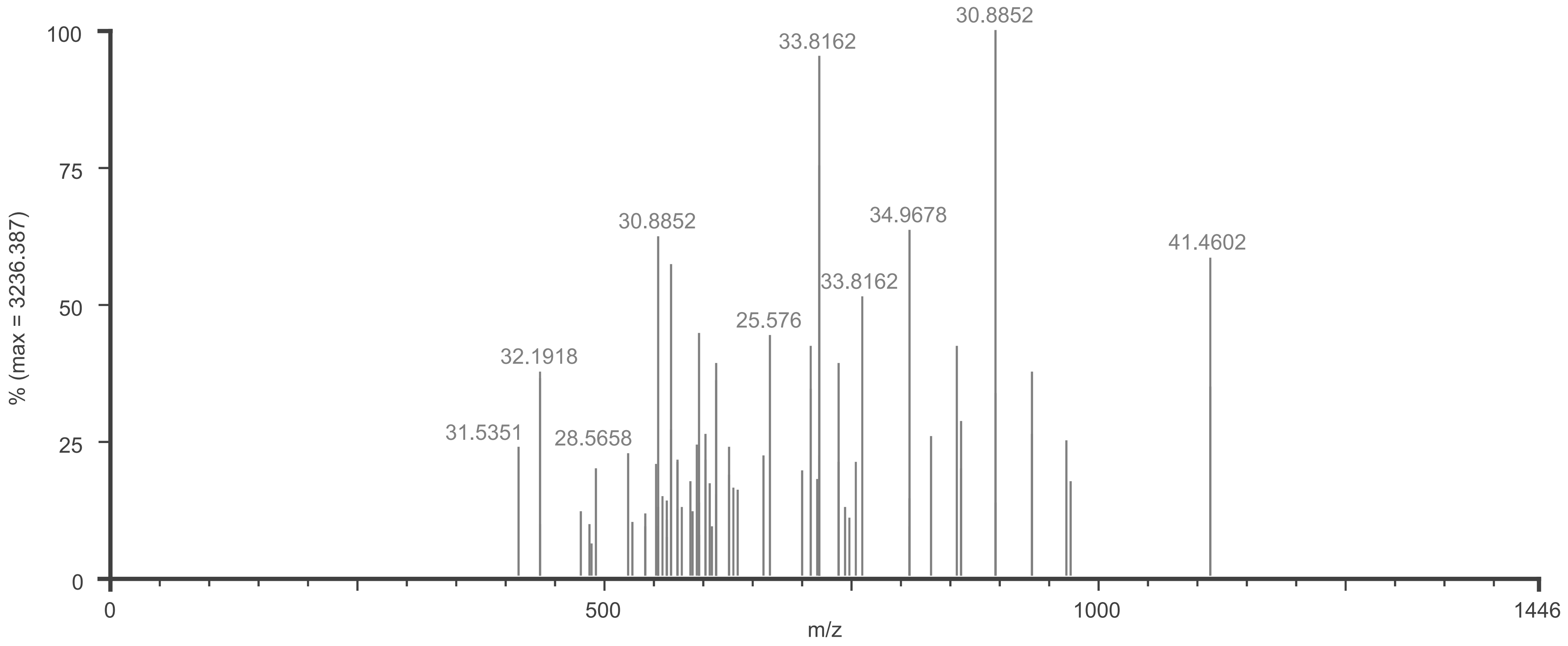

Dr Chris Bagley chris.bagley@imvs.sa.gov.au (08) 8222 3714
Dr Ian Milne   ian.milne@imvs.sa.gov.au (08) 8222 3716

Protein Match Details

Sample:	*C:\MS Files\20-1-06\06-006-1.raw*
Accession:	*37999050*
Name:	*37999050*
Description:	*essential protein for meiotic synapsis Oryza sativa japonica cultiv*
Confidence:	*100.0*
Coverage:	*7.377*
Matches:	*5*
Score:	*14.7098*

37999050 Coverage Map

1	MVMAQKTK**EA**	**EITEQDSLLL**	**TR**NLLR**IAIY**	**NISYIR**GLFP	EKYFNDKSVP
51	ALEMKIKKLM	PMDTESRRLI	DWMEKGVYDA	LQKKYLKTLL	FCICEKEEGP
101	MIEEYAFSFS	YPNTSGDEVA	MNLSRTGSKK	NSATFK**SNAA**	**EVTPDQMR**SS
151	ACKMIRTLVS	LMR**TLDQMPE**	**ER**TILMKLLY	YDDVTPEDYE	PPFFKCCADN
201	EAINIWNKNP	LKMEVGNVNS	KHLVLALKVK	SVLDPCDDNN	VNSEDDNMSL
251	DNESDQDNDF	SDTEVRPSEA	ERYIVAPNDG	TCKGQNGTIS	EDDTQDPVHE
301	EELTAQVREW	ICSRDTESLE	VSDVLVNFPD	ISMEMVEDIM	ERLLKDGLLS
351	RAKKDSYSVN	KIADPTTPHI	KKEVIMQNVS	PTEGTKNSNG	DLMYMKALYH
401	ALPMDYVSVG	KLHGKLDGEA	SQNMVRKLIE	KMVQDGYVKN	SANRRLGKAV
451	IHSEVTNRKL	LEIKKILEVD	IAEQMAIDTN	AEPGEPERKD	HLSGHEMRDG
501	STMGCLQSVG	SDLTRTRELP	EPQQNVSMQS	GQEASTVDKD	PSRTPTSVRE
551	QASVCSLESG	VLGQKVRKSL	AGAGGTQCSQ	DKRFRKASTV	KEPILQYVKR
601	QKSQVQVQVQ				

Dr Chris Bagley chris.bagley@imvs.sa.gov.au (08) 8222 3714

Dr Ian Milne   ian.milne@imvs.sa.gov.au (08) 8222 3716

Peptide Match # 1

Sequence:	*EAEITEQDSLLLTR*
Score:	*220.5093*
Mass:	*1616.8258*
Modifications:	*None*

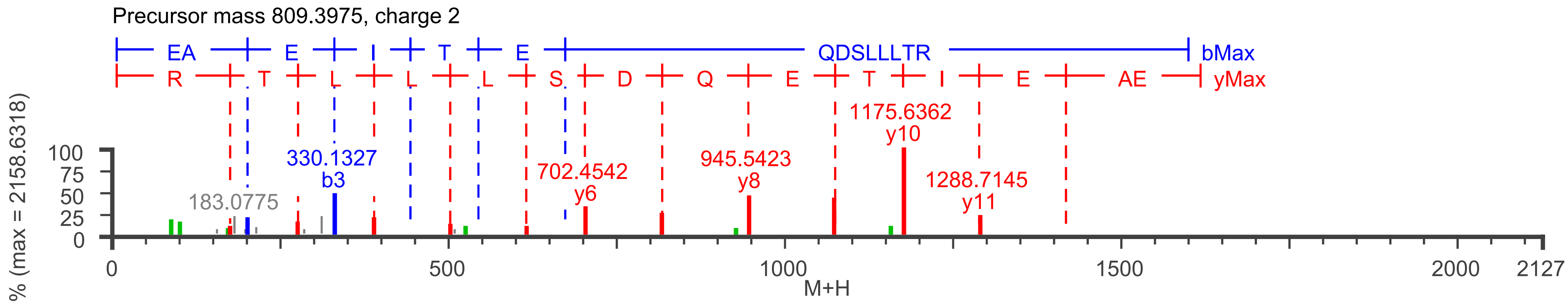

a	102.055	173.093	302.135	415.219	516.267	645.31	773.368	888.395	975.427	1088.511	1201.595	1314.679	1415.727	--
	(0.001)	(0.002)	(-0.003)	(0.001)	(0.001)									
b	130.05	201.088	330.13	443.214	544.262	673.304	801.363	916.39	1003.422	1116.506	1229.59	1342.674	1443.722	--
		(0)	(-0.003)	(0.004)	(-0.004)	(0.017)								
	E	A	E	I	T	E	Q	D	S	L	L	L	T	R
y''	--	1488.791	1417.754	1288.711	1175.627	1074.58	945.537	817.478	702.451	615.419	502.335	389.251	276.167	175.119
			(0.01)	(-0.003)	(-0.009)	(-0.011)	(-0.005)	(0.002)	(-0.003)	(0.024)	(-0.004)	(-0.005)	(-0.002)	(0.001)
z	--	1471.765	1400.727	1271.685	1158.601	1057.553	928.51	800.452	685.425	598.393	485.309	372.225	259.141	158.093
				(-0.001)	(-0.004)	(0.004)	(-0.015)	(0.013)	(0.083)	(0.027)	(0.025)	(-0.01)	(0.016)	

Dr Chris Bagley chris.bagley@imvs.sa.gov.au (08) 8222 3714

Dr Ian Milne ian.milne@imvs.sa.gov.au (08) 8222 3716

Peptide Match # 2

Sequence: *SNAAEVTPDQMR*

Score: *106.0126*

Mass: *1333.5931*

Modifications: *Hydroxyl DKNP (9)*

Precursor mass 667.7844, charge 2

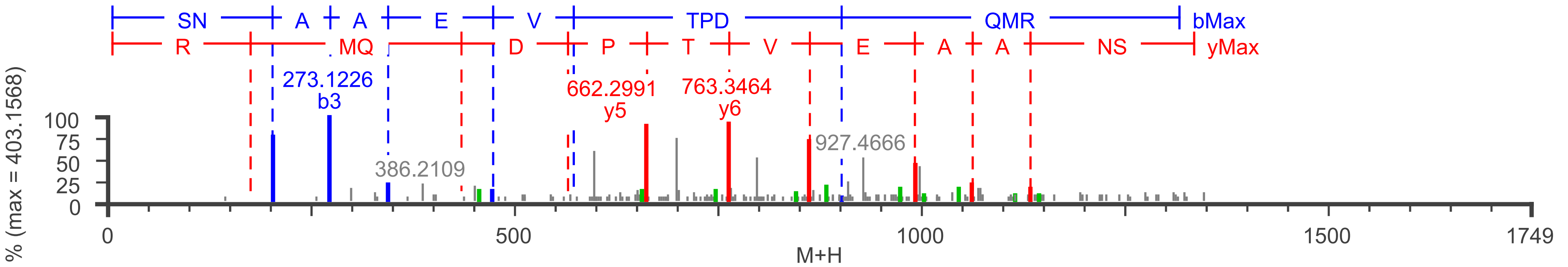

a	*60.045*	*174.088*	*245.125*	*316.162*	*445.205*	*544.273*	*645.321*	*742.373*	*873.395*	*1001.454*	*1132.495*	*--*
	(0.001)			*(-0.003)*	*(-0.002)*	*(0.004)*	*(0.044)*			*(0.024)*		
b	*88.04*	*202.083*	*273.12*	*344.157*	*473.2*	*572.268*	*673.316*	*770.368*	*901.39*	*1029.449*	*1160.489*	*--*
		(0.001)	*(-0.003)*	*(-0.005)*	*(-0.006)*	*(-0.002)*			*(-0.049)*			
	S	*N*	*A*	*A*	*E*	*V*	*T*	*P*	*D*	*Q*	*M*	*R*
y''	*--*	*1247.569*	*1133.526*	*1062.489*	*991.452*	*862.409*	*763.341*	*662.293*	*565.24*	*434.219*	*306.16*	*175.119*
			(-0.024)	*(-0.005)*	*(-0.006)*	*(-0.003)*	*(-0.006)*	*(-0.006)*	*(0.011)*	*(0.008)*		*(0.002)*
z	*--*	*1230.542*	*1116.5*	*1045.462*	*974.425*	*845.383*	*746.314*	*645.267*	*548.214*	*417.192*	*289.133*	*158.093*
				(-0.071)	*(-0.004)*	*(0.023)*	*(0.019)*	*(-0.011)*				

Dr Chris Bagley chris.bagley@imvs.sa.gov.au (08) 8222 3714

Dr Ian Milne ian.milne@imvs.sa.gov.au (08) 8222 3716

Peptide Match # 3

Sequence:	*IAIYNISYIR*
Score:	*86.9647*
Mass:	*1224.6866*
Modifications:	*None*

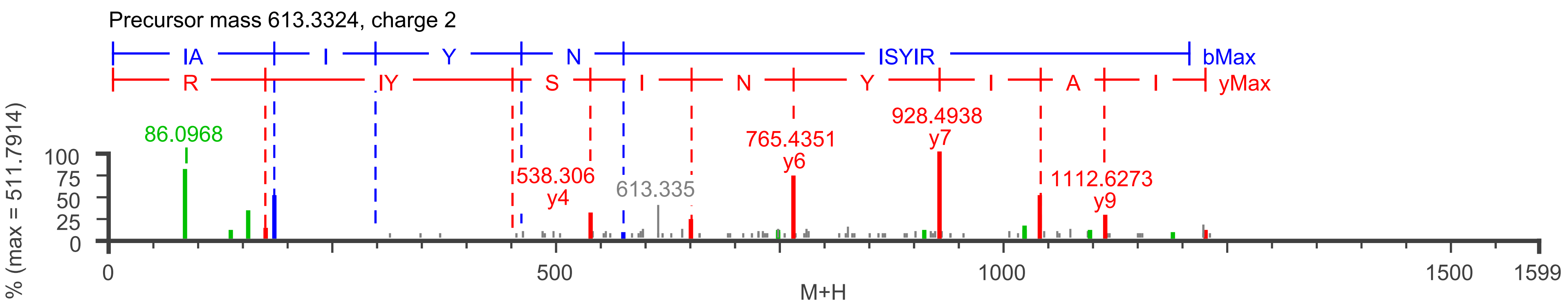

a	*86.097*	*157.134*	*270.218*	*433.281*	*547.324*	*660.408*	*747.44*	*910.504*	*1023.588*	*--*
	(0)	*(0.002)*		*(0.095)*			*(0.029)*	*(0.051)*	*(0.078)*	
b	*114.092*	*185.129*	*298.213*	*461.276*	*575.319*	*688.403*	*775.435*	*938.499*	*1051.583*	*--*
		(-0.002)	*(0.007)*	*(0.022)*	*(-0.062)*					
	I	*A*	*I*	*Y*	*N*	*I*	*S*	*Y*	*I*	*R*
y''	*--*	*1112.61*	*1041.573*	*928.489*	*765.426*	*651.383*	*538.299*	*451.267*	*288.204*	*175.119*
		(-0.017)	*(-0.01)*	*(-0.005)*	*(-0.009)*	*(-0.015)*	*(-0.007)*	*(-0.004)*		*(-0.007)*
z	*--*	*1095.584*	*1024.547*	*911.463*	*748.399*	*634.356*	*521.272*	*434.24*	*271.177*	*158.093*
		(0.012)	*(0.008)*	*(-0.009)*	*(-0.011)*	*(0.076)*	*(0.099)*		*(0.03)*	*(0)*

Dr Chris Bagley chris.bagley@imvs.sa.gov.au (08) 8222 3714

Dr Ian Milne ian.milne@imvs.sa.gov.au (08) 8222 3716

Peptide Match # 4

**Sequence:**	*SNAAEVTPDQMR*
**Score:**	*26.6958*
**Mass:**	*1317.5984*
**Modifications:**	*None*

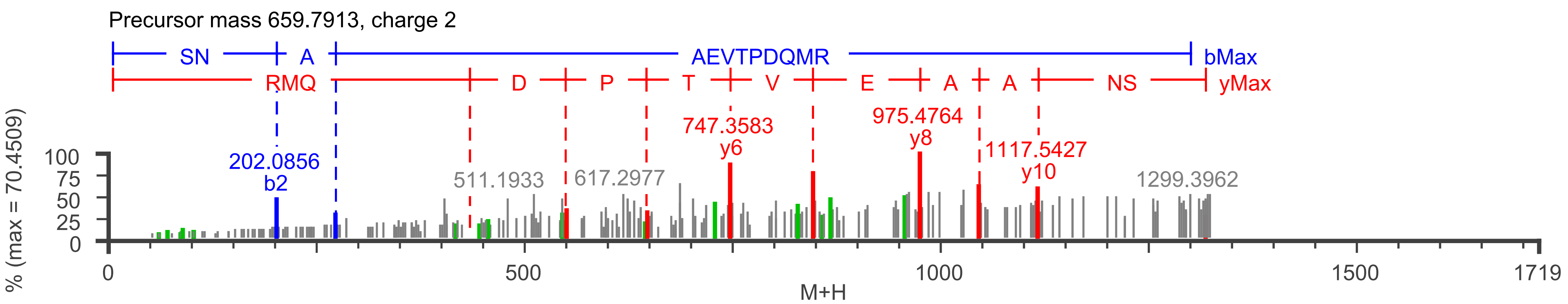

a	60.045	174.088	245.125	316.162	445.205	544.273	645.321	742.373	857.4	985.459	1116.5	--
	(0.008)				(0.074)	(0.003)	(-0.006)		(0.059)			
b	88.04	202.083	273.12	344.157	473.2	572.268	673.316	770.368	885.395	1013.454	1144.494	--
		(-0.003)	(-0.001)									
	S	N	A	A	E	V	T	P	D	Q	M	R
y''	--	1231.574	1117.531	1046.494	975.457	846.414	747.346	646.298	549.246	434.219	306.16	175.12
			(-0.011)	(-0.016)	(-0.019)	(0.002)	(-0.012)	(-0.016)	(0.025)	(0)		
z	--	1214.548	1100.505	1029.468	958.43	829.388	730.319	629.272	532.219	417.192	289.134	158.093
										(0.021)		

Dr Chris Bagley chris.bagley@imvs.sa.gov.au (08) 8222 3714

Dr Ian Milne ian.milne@imvs.sa.gov.au (08) 8222 3716

Peptide Match # 5

Sequence:	*TLDQMPEER*
Score:	*25.8123*
Mass:	*1117.5074*
Modifications:	*None*

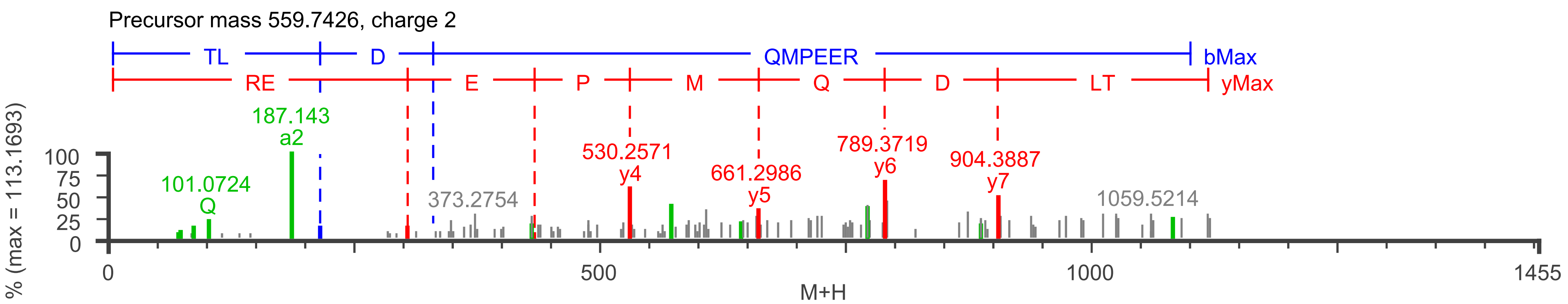

a	*74.061*	*187.145*	*302.172*	*430.23*	*561.271*	*658.323*	*787.366*	*916.409*	--
	(-0)	*(0.002)*		*(-0.028)*					
b	*102.056*	*215.14*	*330.167*	*458.225*	*589.266*	*686.318*	*815.361*	*944.404*	--
		(-0.001)	*(-0.004)*						
	T	*L*	*D*	*Q*	*M*	*P*	*E*	*E*	*R*
y''	--	*1017.468*	*904.383*	*789.357*	*661.298*	*530.257*	*433.205*	*304.162*	*175.12*
			(-0.005)	*(-0.015)*	*(-0.001)*	*(0)*	*(-0.085)*	*(-0.005)*	
z	--	*1000.441*	*887.357*	*772.33*	*644.271*	*513.231*	*416.178*	*287.136*	*158.093*
			(-0.004)	*(-0.034)*	*(-0.062)*				

Dr Chris Bagley chris.bagley@imvs.sa.gov.au (08) 8222 3714

Dr Ian Milne ian.milne@imvs.sa.gov.au (08) 8222 3716
